# A chimeric mRNA vaccine of S-RBD with HA conferring broad protection against influenza and COVID-19 variants

**DOI:** 10.1371/journal.ppat.1012508

**Published:** 2024-09-20

**Authors:** Tianjiao Hao, Yulei Li, Peipei Liu, Xi Wang, Ke Xu, Wenwen Lei, Ying Li, Rong Zhang, Xiaoyan Li, Xin Zhao, Kun Xu, Xuancheng Lu, Yuhai Bi, Hao Song, Guizhen Wu, Baoli Zhu, George F. Gao

**Affiliations:** 1 CAS Key Laboratory of Pathogen Microbiology and Immunology, Institute of Microbiology, Chinese Academy of Sciences, Beijing, People’s Republic of China; 2 University of Chinese Academy of Sciences, Beijing, People’s Republic of China; 3 Clinicopathological Diagnosis & Research Center, the Affiliated Hospital of Youjiang Medical University for Nationalities, Baise, People’s Republic of China; 4 Key Laboratory of Tumor Molecular Pathology of Guangxi Higher Education Institutes, Baise, People’s Republic of China; 5 NHC Key Laboratory of Biosafety, National Institute for Viral Disease Control and Prevention, Chinese Center for Disease Control and Prevention, Beijing, People’s Republic of China; 6 State Key Laboratory for Conservation and Utilization of Subtropical Agro-Bioresources, Guangxi University, Nanning, People’s Republic of China; 7 National Key Laboratory of Intelligent Tracking and Forecasting for Infectious Diseases (NITFID), Chinese Center for Disease Control and Prevention, Beijing, People’s Republic of China; 8 Research Network of Immunity and Health (RNIH), Beijing Institutes of Life Science, Chinese Academy of Sciences, Beijing, People’s Republic of China; 9 Beijing Key Laboratory of Emerging Infectious Diseases, Institute of Infectious Diseases, Beijing Ditan Hospital, Capital Medical University, Beijing People’s Republic of China; 10 Beijing Institute of Infectious Diseases, Beijing, People’s Republic of China; 11 Department of Pathogenic Biology, School of Basic Medical Sciences, Southwest Medical University, Luzhou, People’s Republic of China; Icahn School of Medicine at Mount Sinai, UNITED STATES OF AMERICA

## Abstract

Influenza and coronavirus disease 2019 (COVID-19) represent two respiratory diseases that have significantly impacted global health, resulting in substantial disease burden and mortality. An optimal solution would be a combined vaccine capable of addressing both diseases, thereby obviating the need for multiple vaccinations. Previously, we conceived a chimeric protein subunit vaccine targeting both influenza virus and severe acute respiratory syndrome coronavirus 2 (SARS-CoV-2), utilizing the receptor binding domain of spike protein (S-RBD) and the stalk region of hemagglutinin protein (HA-stalk) components. By integrating the S-RBD from the SARS-CoV-2 Delta variant with the headless hemagglutinin (HA) from H1N1 influenza virus, we constructed stable trimeric structures that remain accessible to neutralizing antibodies. This vaccine has demonstrated its potential by conferring protection against a spectrum of strains in mouse models. In this study, we designed an mRNA vaccine candidate encoding the chimeric antigen. The resultant humoral and cellular immune responses were meticulously evaluated in mouse models. Furthermore, the protective efficacy of the vaccine was rigorously examined through challenges with either homologous or heterologous influenza viruses or SARS-CoV-2 strains. Our findings reveal that the mRNA vaccine exhibited robust immunogenicity, engendering high and sustained levels of neutralizing antibodies accompanied by robust and persistent cellular immunity. Notably, this vaccine effectively afforded complete protection to mice against H1N1 or heterosubtypic H5N8 subtypes, as well as the SARS-CoV-2 Delta and Omicron BA.2 variants. Additionally, our mRNA vaccine design can be easily adapted from Delta RBD to Omicron RBD antigens, providing protection against emerging variants. The development of two-in-one vaccine targeting both influenza and COVID-19, incorporating the mRNA platform, may provide a versatile approach to combating future pandemics.

## Author summary

Developing a combined vaccine that can protect against both influenza and COVID-19 would be highly advantageous, considering the significant disease burden caused by these respiratory viruses. In our study, we designed an mRNA vaccine candidate that incorporates a chimeric antigen composed of components from the SARS-CoV-2 Delta variant and H1N1 influenza virus. This vaccine demonstrated strong immunogenicity, inducing robust and sustained levels of neutralizing antibodies and cellular immunity in mouse models. Importantly, it offered complete protection against homologous and heterologous influenza virus strains, as well as the Delta variant and Omicron BA.2 variant of SARS-CoV-2. This research highlights the potential and versatility of an mRNA-based two-in-one vaccine approach, providing a valuable strategy against future infection caused by influenza virus and SARS-CoV-2.

## Introduction

Coronavirus disease 2019 (COVID-19), caused by severe acute respiratory syndrome coronavirus 2 (SARS-CoV-2), has had an immeasurable impact on global health, the economy, and social stability. Large-scale vaccinations are critical to fight against COVID-19 and control the pandemic. After four years of global efforts, several COVID-19 vaccines have been released with proven efficacy in Phase 3 clinical trials, and approved for use in many countries, including mRNA vaccines, inactivated vaccines, adenovirus vectored vaccines and protein subunit vaccines [1–5]. Unfortunately, the effectiveness of the current vaccines has been challenged by the ongoing spread of some highly transmissible variants of concern (VOCs), including the Alpha, Beta, Gamma, Delta and Omicron variants, and their ability to cause breakthrough infections.

Influenza virus causes a substantial global health and economic burden despite the availability of commercial vaccines. Current seasonal vaccines primarily elicit antibodies that target immunodominant hypervariable epitopes in the head domain of the hemagglutinin (HA) glycoprotein. This results in conventional vaccine effectiveness ranging from 10% to 60% and necessitates the need for seasonal updates of virus strains included in licensed vaccines [6,7]. In addition, sporadic human cases infected by avian influenza viruses (AIVs), including H7N9, H7N4, H5N1, H5N6, H5N8, H10N3, and H3N8 subtypes, were also reported [7–12]. The seasonal influenza epidemics and occasional zoonotic influenza virus infections in humans have become a significant global disease burden of great concern.

In response to two viral pandemics, we reasonably designed a chimeric protein subunit vaccine against influenza and COVID-19 with S-RBD and HA-stalk [13]. The S-RBD from SARS-CoV-2 Delta variant was fused with the headless HA from H1N1 influenza virus (H1Delta), which can form trimers in solution. Cryo-EM structure of the chimeric protein complexed with the RBD-targeting CB6 [14] and the HA-stalk-targeting CR9114 [15] antibodies shows that the trimeric protein is stable and accessible for binding of neutralizing antibodies. Immunization of the vaccine elicited high and long-lasting neutralizing antibodies, and effectively protected mice against the challenges of lethal H1N1 or heterosubtypic H5N8, and Delta or Omicron variants.

mRNA-based vaccines are attractive platforms for prophylactic emerging infectious disease vaccine candidates because of their unique advantages, including rapid large-scale production, strong immunogenicity in humoral and cellular immunity, and safety [16–18]. In this study, we developed an LNP-encapsulated chemically modified mRNA vaccine candidate that encodes the HA-stalk and S-RBD chimeric antigen, the antigen designed as we did for the protein submit vaccine earlier [13]. We found that the mRNA vaccine induced both robust humoral and cellular responses. Remarkably, this vaccine delivered unequivocal and comprehensive protection to mice, effectively guarding against lethal H1N1 and heterosubtypic H5N8 strains, as well as the Delta variant and the Omicron BA.2 variant. Additionally, our mRNA vaccine design can be easily adapted from Delta RBD to Omicron RBD antigens, providing protection against emerging variants. Together, the data suggest that mRNA/LNP vaccines expressing chimeric antigens hold promise for efficacy across a broad spectrum of influenza A viruses (IAVs) and SARS-CoV-2 variants.

## Results

### Construction and characterization of mRNA vaccine

**Fig 1:**
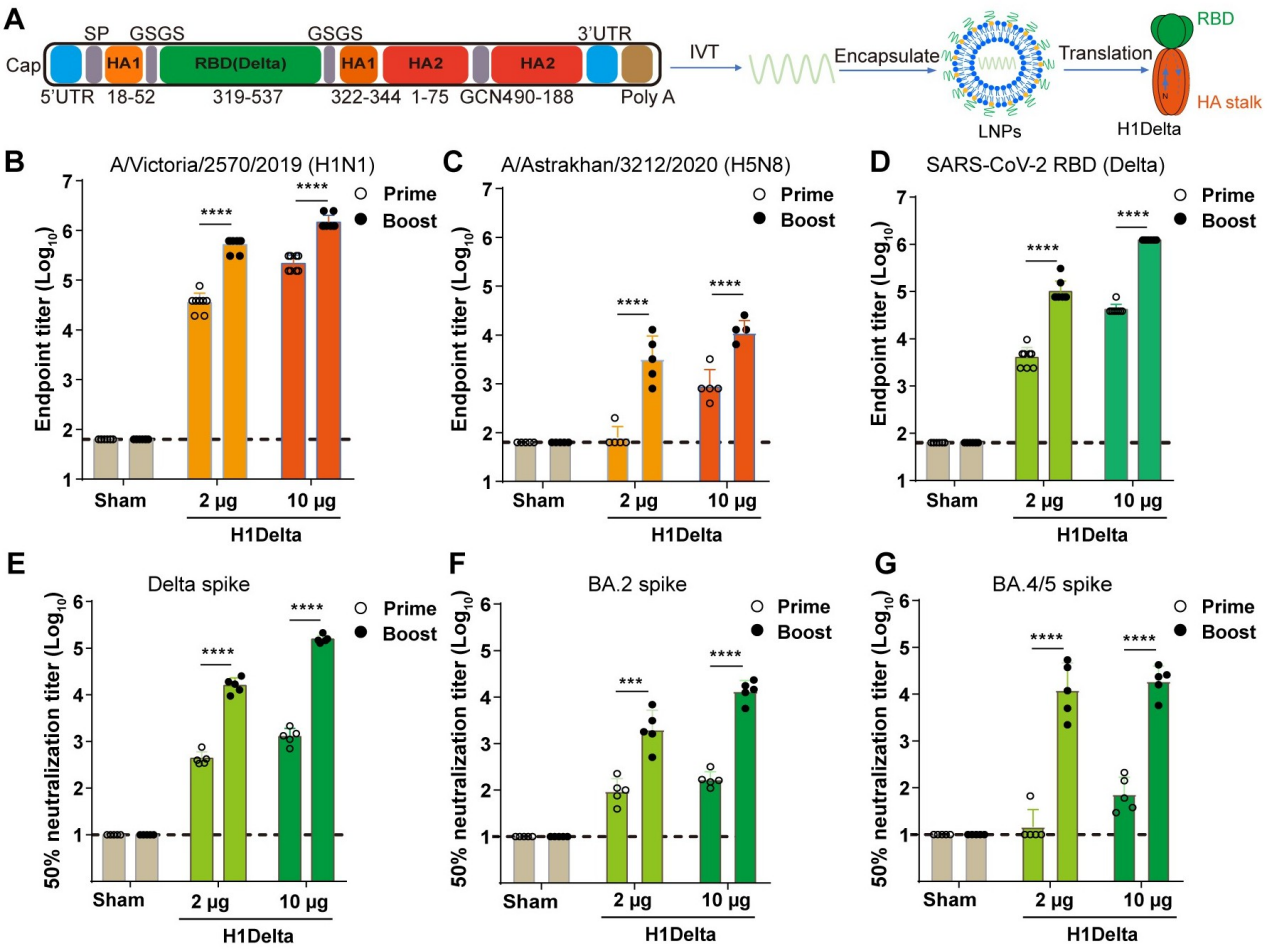
H1Delta mRNA vaccine design and immunogenicity in mice. (A) Schematic of the H1Delta mRNA vaccine design. The mRNA transcript includes a 5’ UTR, the H1Delta coding sequence [13], a 3’ UTR, and a poly-A tail, all synthesized from linear DNA templates. SP, signal peptide. For detailed descriptions of the mutation sites, please see the Materials and Methods section. (B-G) Groups of 6- to 8-week-old female BALB/c mice (n = 5) were vaccinated with two doses of H1Delta mRNA vaccine containing either 2 or 10 μg of immunogens at 3-week intervals. Serum samples were collected 19 and 35 days after the initial immunization for ELISA and neutralization assays. (B-D) H1 HA (B), H5 HA (C) and SARS-CoV-2 Delta RBD (D) specific IgG titers are determined by ELISA. (E-G) Neutralization assay results for sera from mice immunized with H1Delta mRNA. 50% neutralization titers of pseudovirus against Delta (E), BA.2 (F), and BA.4/5 (G) are shown. Limit of detections (LODs) were indicated by dashed lines.

We designed the mRNA vaccine with the stalk region of HA protein from A/Victoria/2570/2019 (H1N1) as the backbone, and the RBD from SARS-CoV-2 Delta variants spike protein replaced the head region of HA, similar to the H1Delta protein vaccine [13]. Additionally, we incorporated the 5’ untranslated region (UTR) and 3’ ployA in the mRNA construction. To enhance mRNA translation *in vivo* and prevent innate immune sensing, the mRNA contains the modified nucleoside N1-methylpseudouridine [19] (Fig 1A). Both culture supernatants and cell lysates from H1Delta-mRNA-transfected cells exhibited reactivity with a SARS-CoV-2 RBD-specific antibody, confirming the successful secreted expression of the target proteins at a molecular weight of approximately 70 kDa (predicted molecular weight is 53 kDa) (S1A Fig). This observed higher molecular weight is due to glycosylation, which has been reported in both HA and RBD proteins [20,21]. Proteins in the cell lysate are often incompletely glycosylated, whereas secreted proteins are fully glycosylated, resulting in a higher molecular weight. The mRNA and lipid nanoparticle (LNP) complex formulations were prepared using a modified procedure, as previously described for small interfering RNA (siRNA) [22]. The encapsulation efficacy of mRNA-RBD was greater than 95% (96.8%), as determined by the Quant-iT RiboGreen fluorescence assay (S1D Fig). Dynamic light scattering analysis of LNPs in Dulbecco’s phosphate-buffered saline indicated an average particle size of 82 nm (S1B Fig). Cryo-electron microscopy analysis further revealed that H1Delta mRNA particles exhibited a uniform solid spherical morphology (S1C Fig).

### Immunogenicity of H1Delta mRNA vaccine in mice

To assess the immunogenicity of the H1Delta mRNA vaccine against influenza and COVID-19, BALB/c mice were immunized twice, 3 weeks apart, with 2 or 10 μg doses; empty LNPs were used as control. Serum samples were collected 19 and 35 days after initial vaccination. For influenza virus HA-specific antibodies, a rapid increase in H1-specific IgG antibodies was observed in mice following the second immunization with either 2 or 10 μg of H1Delta mRNA-LNP, reaching approximately10^6^. In contrast, no IgG antibodies were detected in the sera of mice immunized with empty LNPs (Fig 1B). Following the boost, mice that received 10 μg H1Delta mRNA-LNP exhibited cross-reactive H5-specific IgG, reaching an endpoint titer of ~10^4^ (Fig 1C). In terms of SARS-CoV-2, remarkably, a second immunization with either 2 or 10 μg of H1Delta mRNA vaccine resulted in a rapid elevation of IgG and neutralizing antibodies in mice. The RBD-specific IgG induced by 10 μg H1Delta booster vaccination in mice reached endpoint titer approximately 10^6^ (Fig 1D). Moreover, the serological neutralizing antibody titers (GMT) against pseudotyped virus displaying Delta, Omicron (BA.2), and Omicron (BA.4/5) spike proteins reached ~154882, ~13366 and ~18750, respectively (Fig 1E, 1F and 1G). We then conducted a detailed analysis of the immune response induced by the H1Delta mRNA vaccine, focusing on different IgG subtypes (S2 Fig). Specifically, the ELISA results showed high levels of H1-specific IgG1 and IgG2a (S2B and S2C Fig), as well as SARS-CoV-2 Delta RBD-specific IgG1 and IgG2a titers (S2E and S2F Fig). The presence of both IgG1 and IgG2a indicates a balanced Th1/Th2 immune response, as IgG1 is typically associated with Th2 responses while IgG2a is associated with Th1 responses. This balanced Th1/Th2 response is crucial for effective immunity, as Th1 responses promote cell-mediated immunity, while Th2 responses support humoral immunity. These results serve to underscore the performance of the H1Delta mRNA vaccine in eliciting a broad immune response.

**Fig 2:**
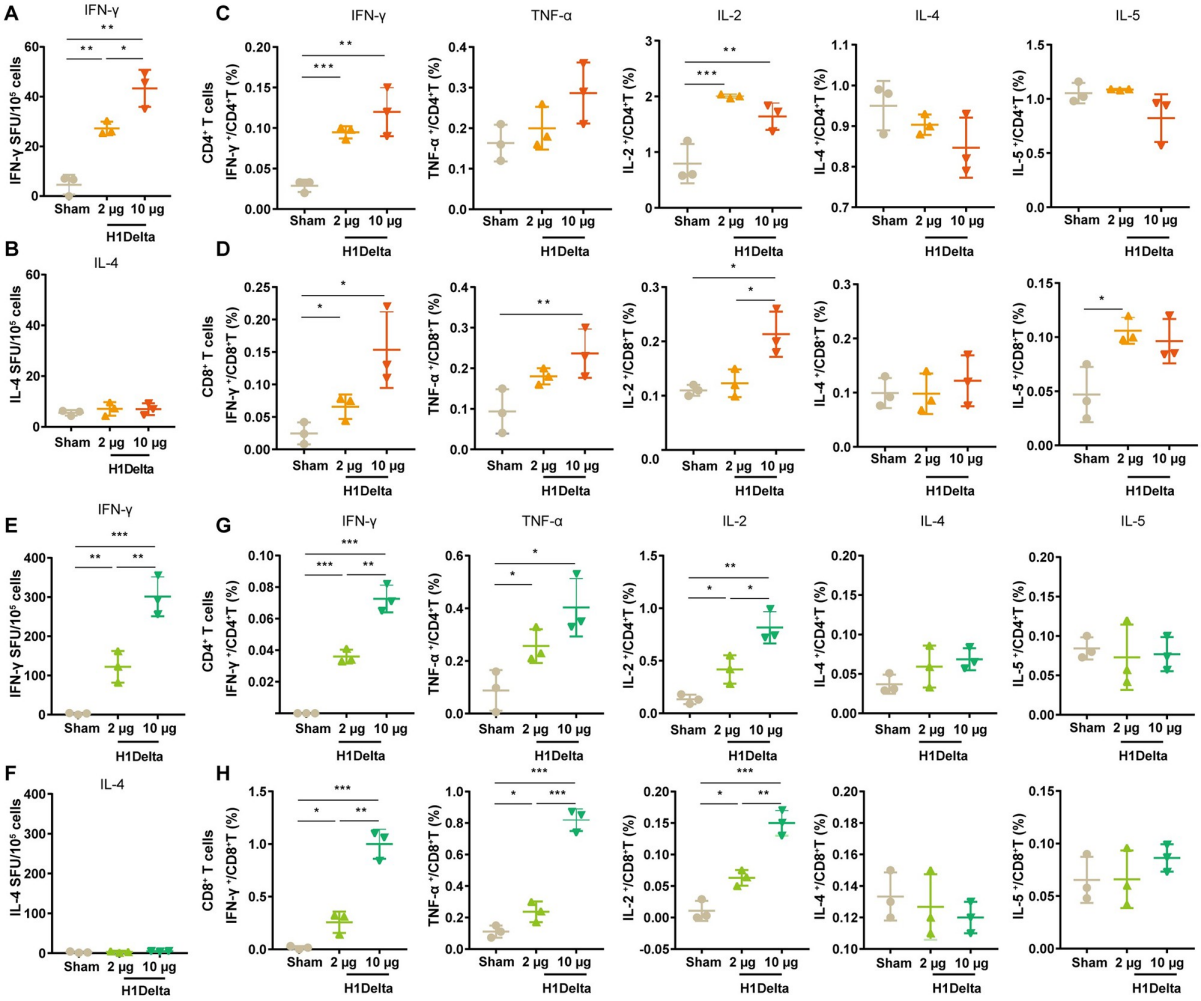
H1Delta mRNA vaccine elicits robust cellular immune responses in mice. Female BALB/c mice (n = 3) were vaccinated with 2 or 10 μg doses of H1Delta mRNA-LNP or empty LNP. Spleens were collected 120 days after the initial immunization. An ELISpot assay was performed to evaluate the capacity of splenocytes to secrete IFN-γ (A) and IL-4 (B) following re-stimulation with H1 stalk peptide pools. A flow cytometry with ICS assay was conducted to quantify the proportions of s CD8^+^ (C) and CD4^+^ (D) T cells secreting IFN-γ^+^, TNF-α^+^, IL-2^+^, IL-4^+^ or IL-5^+^ following re-stimulation with H1 stalk peptide pools. Another portion of the splenocytes was stimulated with SARS-CoV-2 RBD peptide pool to detect cytokine production. This was measured using ELISpot shown in (E) and (F), and ICS shown in (G) and (H).

To characterize the long-lasting cellular immune response induced by H1Delta mRNA-LNP, enzyme-linked immunospot (ELISpot) and intracellular cytokine staining (ICS) assays were performed. Spleens of BALB/c mice were harvested 120 days after immunization. Re-stimulation with H1 stalk peptide pools *in vitro*, interferon-γ (IFN-γ) spot-forming units per million splenocytes were 27 ± 1.6 and 43 ± 4.2 (mean ± SEM) in mice inoculated with 2 μg and 10 μg of H1Delta mRNA-LNP, respectively, significantly higher than those in sham group mice, while interleukin-4 (IL-4) spot-forming units were slightly above the background (Fig 2A and 2B and S2A Fig). Meanwhile, we analyzed cytokine-producing CD4^+^ and CD8^+^ T cells in splenocytes through flow cytometry with ICS. CD4^+^ T cells exhibited increased percentages of IFN-γ, and interleukin-2 (IL-2) producing cells, with very low percentages of IL-4 and interleukin-5 (IL-5) producing cells (Fig 2C, S3C and S3D Fig). Among CD8^+^ T cells, the percentages of cells secreting IFN-γ, tumor necrosis factor alpha (TNF-α), and IL-2 were significantly increased compared with the sham group vaccination, while there was no significant difference in IL-4 secreting cells (Fig 2D, S3C and S3D Fig).

Following re-stimulation with SARS-CoV-2 RBD peptide pools *in vitro*, IFN-γ spot-forming units per million splenocytes were 122 ± 23 and 301 ± 29 (mean ± SEM), respectively, significantly higher compared to sham group, whereas IL-4 spot-forming units were few above the background, indicating a strong Th1 response (Fig 2E and 2F and S3B Fig). RBD protein-specific CD4^+^ and CD8^+^ T cell responses were evaluated by ICS. H1Delta mRNA-LNP elicited antigen-specific, multifunctional CD4^+^ and CD8^+^ T cells expressing Th1 immune response cytokines (IFN-γ, TNF-α and IL-2) significantly increased (Fig 2G and 2H, S3E and S3F Fig). These results indicate that H1Delta vaccination induced robust Th1-biased T cell responses against SARS-CoV-2.

### Protection efficacy to homologous H1N1 and heterologous H5N8 influenza virus of H1Delta vaccine in mice

**Fig 3:**
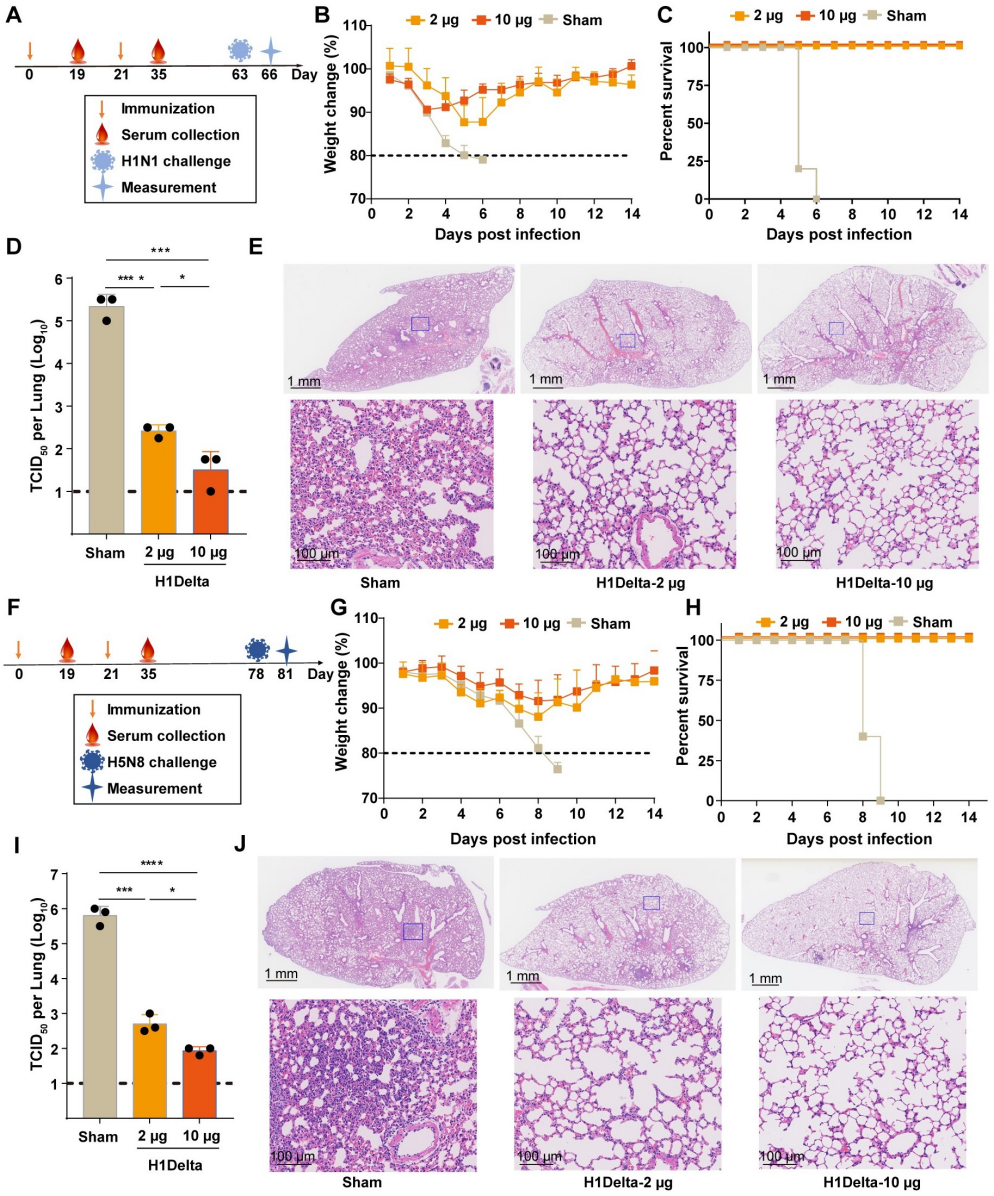
Protection efficacy of H1Delta mRNA vaccine against homologous H1N1 and heterologous H5N8. (A-D) Groups of 6- to 8-week-old female BALB/c mice (n = 8) were vaccinated with two doses of the H1Delta mRNA vaccine, containing either 2 or 10 μg immunogen, at 3-week intervals (A). Serum samples were collected 19 and 35 days after initial immunization. Mice were intranasally challenged with 20 × mLD_50_ of A/Brisbane/02/2018 (H1N1) virus. Vaccine efficacy was assessed by measuring morbidity (weight loss) (B), mortality (survival) (C) in five mice. Lung tissues from the other 3 mice were harvested and split to test lung viral titers on 3 DPI (D), and histological pathology analyses (E). (F-J) Groups of 6- to 8-week-old female BALB/c mice (n = 8) were vaccinated with two doses of the H1Delta mRNA vaccine, containing either 2 or 10 μg immunogen, at 3-week intervals (F). Serum samples were collected 19 and 35 days after initial immunization. Mice were intranasally challenged with 10 × mLD_50_ of reassortment A/Astrakhan/3212/2020(H5N8) virus. Vaccine efficacy was assessed by measuring morbidity (weight loss) (G) and mortality (survival) (H) in five mice. Lung tissues from the other 3 mice were harvested and split to test lung viral titers on 3 DPI (I), and histological pathology analyses (J). Representative lung sections were stained with H&E. Scale bars are labelled.

To assess the protective efficacy of the H1Delta mRNA vaccine, immunized mice were subjected to intranasal challenge with a lethal dose of 20 × mLD_50_ of A/Brisbane/02/2018 (H1N1) virus (Fig 3A), which shares a 99.42% sequence similarity with A/Victoria/2570/2019 in the HA stalk region. All mice in the sham group died within 6 days, while all the mice in the H1Delta mRNA vaccine immunization group survived (Fig 3B and 3C). Furthermore, vaccination resulted in a significant reduction in viral load in the lung tissue of mice on day 3 post-infection (DPI) (Fig 3D). Histopathological examination of the lung sections showed typical features of moderate-to-severe interstitial pneumonia in control-immunized mice (Fig 3E). In contrast, vaccinated animals exhibited only mild histological changes with reduced pathological severity (Fig 3E).

To further explore the protective efficacy of H1Delta mRNA vaccine against the heterologous H5N8 influenza virus, we conducted intranasal challenges with 10 × mLD_50_ of reassortment A/Astrakhan/3212/2020(H5N8) virus 78 days after the initial vaccination (Fig 3F). Eight days following H5N8 challenge, all control-vaccinated mice reached humane endpoints and were subsequently euthanized. Mice vaccinated with the H1Delta mRNA vaccine exhibited 100% protection from mortality (Fig 3G and 3H). Furthermore, vaccinated mice had lower lung viral loads compared to control-immunized mice (Fig 3I). Subsequent histopathological examination also indicated that the control group had more extensive lung damage, combined lesions and inflammatory cell infiltration in a larger area, than the H1Delta mRNA-immunized mice (Fig 3J). These results suggest that H1Delta mRNA vaccination can effectively protect against heterologous H5N8 influenza virus induced lung injury in mice.

### Protection efficacy to SARS-CoV-2 of H1Delta mRNA vaccine in mice

**Fig 4:**
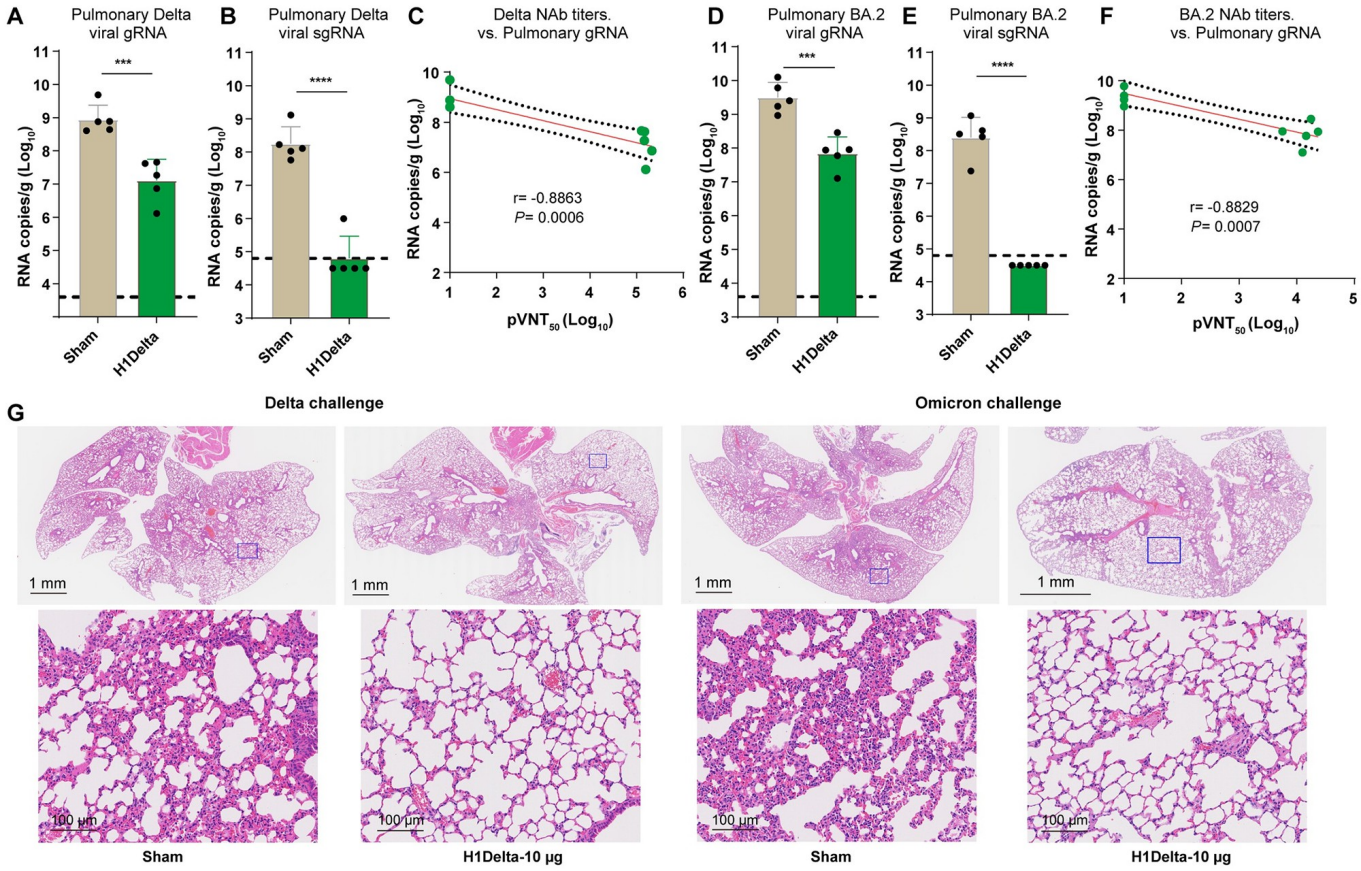
Protection efficacy of H1Delta mRNA vaccine against SARS-CoV-2. (A-C) Groups of 6- to 8-week-old female BALB/c mice (n = 10) were vaccinated with two doses of the H1Delta mRNA vaccine, containing 10 μg immunogen or sham (control), at 3-week intervals. Five mice were randomly selected from each group and challenged with 5 × 10^5^ TCID_50_ of Delta SARS-CoV-2 variant, and (D–F) the other five were challenged with 5 × 10^5^ TCID_50_ of Omicron (BA.2) variant 140 days after the initial immunization. Mice challenged with Delta variant had previously received Ad5-hACE2 intranasally 5 days before the challenge. Mice were euthanized and necropsied on day 3 post-infection, with lung tissues collected for viral titration and pathological analysis. (A) Pulmonary Delta viral gRNA levels were detected by qRT-PCR. (B) Pulmonary Delta viral sgRNA levels were detected by qRT-PCR. (C) Plots show correlations and corresponding two-sided p values between pVNT_50_ of Delta variant and Delta viral gRNA. (D) Pulmonary Omicron viral gRNA levels were detected by qRT-PCR. (E) Pulmonary Omicron viral sgRNA levels were detected by qRT-PCR. (F) Plots show correlations and corresponding two-sided p values between pVNT_50_ of Omicron variant and Omicron viral gRNA. LODs were indicated by dashed lines. (G) Histopathological analyses of lung sections from mice challenged with Delta or Omicron. Shown are the representative lung sections by H&E staining. Scale bars are labelled.

To further explore the protective efficacy of H1Delta mRNA vaccine, BALB/c mice (n = 10) immunized with 10 μg vaccine were randomly divided into two batches (n = 5) and challenged with SARS-CoV-2 Delta and Omicron (BA.2 sub-variant) variants for 5 × 10^5^ TCID_50_, respectively. For the Delta variant challenge, the mice were transduced with adenovirus serotype 5 (Ad5) expressing hACE2 five days before virus inoculation as previously described [23]. At 3 DPI, all mice were euthanized, and lung samples were collected for virus titration. In mice challenged with Delta SARS-CoV-2 variant, high levels of viral genomic RNA (gRNA) (average: 8.71 × 10^8^ copies/g) was detected in control-immunized mice (Fig 4A). By contrast, the averages of pulmonary viral gRNA of vaccine-immunized mice were significantly reduced (average: 1.30 × 10^7^ copies/g) (Fig 4A). The pulmonary viral subgenomic RNA (sgRNA) were detected in all mice in the sham group with high levels (average: 1.76 × 10^8^ copies/g) (Fig 4B), but only detectable in one mouse receiving H1Delta vaccine with the titer of 9.88 × 10^5^ copies/g (Fig 4B). Notably, an analysis of immune correlates of protection following vaccination revealed a strong correlation between neutralizing antibody (NAb) titers and the reduction of pulmonary Delta SARS-CoV-2 gRNA based on a linear model (r = -0.8863, p = 0.0006) (Fig 4C).

For mice challenged with Omicron SARS-CoV-2 variant, the averages of pulmonary viral gRNA were 3.13 × 10^9^ copies/g in the sham group but reduced to 6.97 × 10^7^ copies/g in the H1Delta vaccine group (Fig 4D). Correspondingly, pulmonary viral sgRNA were detected in all mice in the sham group with high levels (average: 2.49 × 10^8^ copies/g), but undetectable in the all mice receiving H1Delta vaccine, suggesting the complete control of Omicron viral replication (Fig 4E). In the context of Omicron SARS-CoV-2 challenge, there was a strong inverse correlation between neutralizing antibody (NAb) titers and pulmonary viral gRNA levels, as demonstrated by a linear model (r = -0.8829, p = 0.0007) (Fig 4F).

The histopathological analysis showed that at 3 days post either Delta or Omicron variant infection, control-immunized mice showed moderate-to-severe histopathological changes in lung, including infiltration of inflammatory cells in the alveolar lumen, pulmonary vascular congestion, and vascular congestion (Fig 4G). In contrast, mice vaccinated with H1Delta vaccine exhibited relieved lung injury (Fig 4G). The histopathology result was consistent with the tendency of pulmonary viral gRNA shown above and demonstrated H1Delta provided strong protection against not only the Delta but also the Omicron variants.

### Duration and long-lasting protection of humoral response induced by H1Delta mRNA vaccine

**Fig 5:**
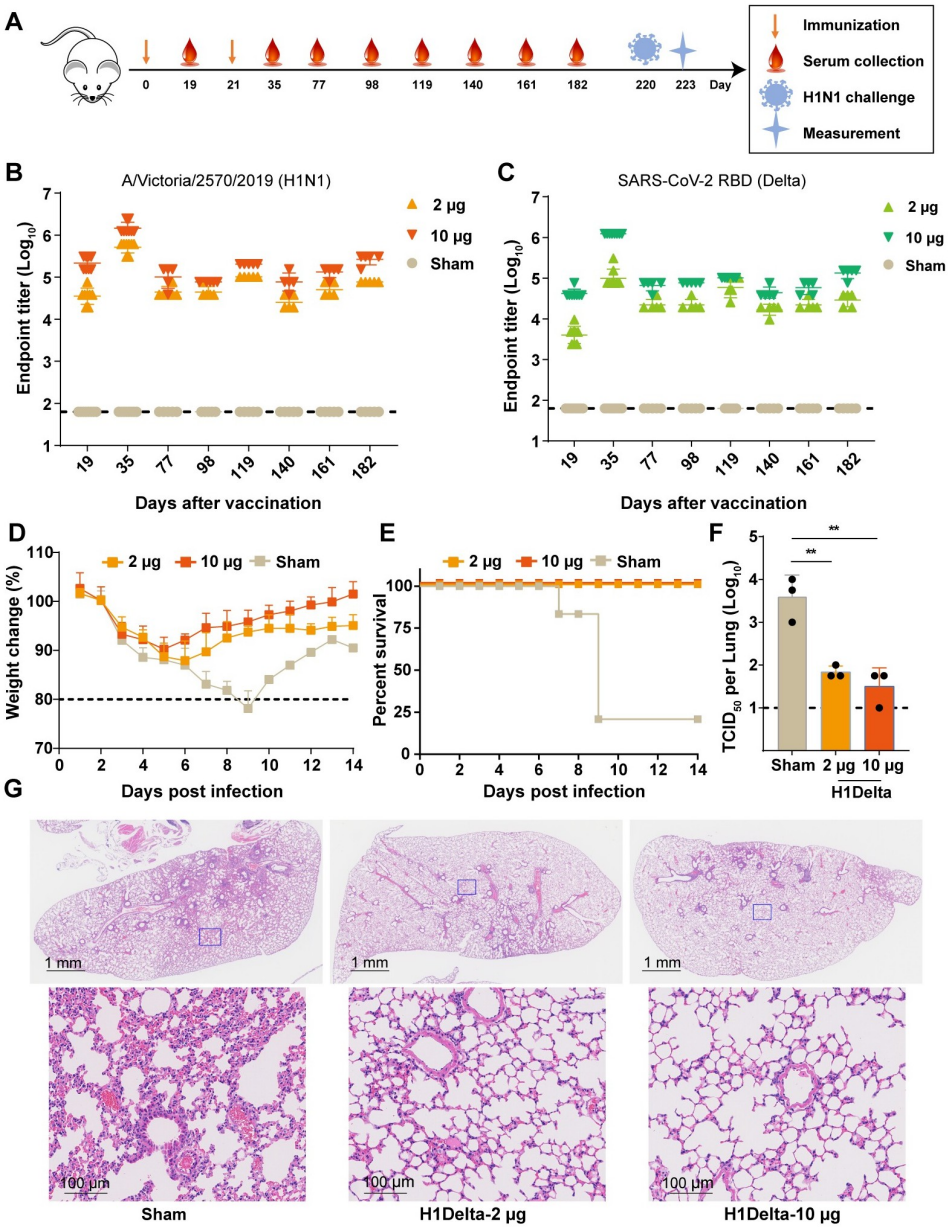
Long-lasting protection of H1Delta mRNA vaccine. (A) Time course of H1Delta vaccine immune antibody monitoring, viral challenge and measurement in mice. Groups of 6- to 8-week-old female BALB/c mice (n = 8) were vaccinated with two doses of the H1Delta mRNA vaccine, containing either 2 or 10 μg immunogen, at 3-week intervals. Specific IgG titers against H1 HA (B) and SARS-CoV-2 Delta RBD (C) are determined by ELISA at the indicated time points. Mice were intranasally challenged with 20 × mLD_50_ of A/Brisbane/02/2018 (H1N1) virus. Vaccine efficacy was assessed by measuring morbidity (weight loss) (D) and mortality (survival) (E) in five mice over a 14-day period following virus infection. Lung tissues from the other three mice were harvested on 3 DPI and split to test lung viral titers (F) and for histological pathology analyses (G). Virus titers in the lungs were detected, with limits of detection (LODs) indicated by dashed lines. Representative lung sections are shown with H&E staining. Scale bars are labelled.

To evaluate the durability and long-term protection of H1Delta, BALB/c mice were bled at designated times to explore the kinetics of induced humoral response (Fig 5A). Time course studies of H1-specific antibodies and RBD-specific antibodies in the serum of vaccinated mice showed that both increased to a peak of approximately 10^6^ at 35 days post-immunization. Impressively, even after 182 days post-immunization, the H1-specific antibody and RBD-specific antibody titers in the 10 μg immunized group were still maintained at about 10^5^ (Fig 5B and 5C). At 220 days post-vaccination, the mice were challenged with 20 × mLD_50_ of A/Brisbane/02/2018 (H1N1) virus. The results showed that the body weights of mice in both the 2 μg and 10 μg groups exhibited a moderate decrease (<12%) at 5 DPI, followed by a gradual recovery. In contrast, the body weights of mice in the sham group continued to decrease, with four out of five mice succumbing to the infection. The single surviving mouse in the sham group began to recover weight starting at 9 DPI (Fig 5D and 5E). Moreover, the lungs of H1Delta-immunized mice showed significantly reduced infectious viral burden (Fig 5F). Histopathological examination revealed severe bronchopneumonia and interstitial pneumonia in sham mice. In contrast, only very mild bronchopneumonia was observed in the H1Delta-immunized mice (Fig 5G).

### Development of H1BA.2 mRNA vaccine

**Fig 6:**
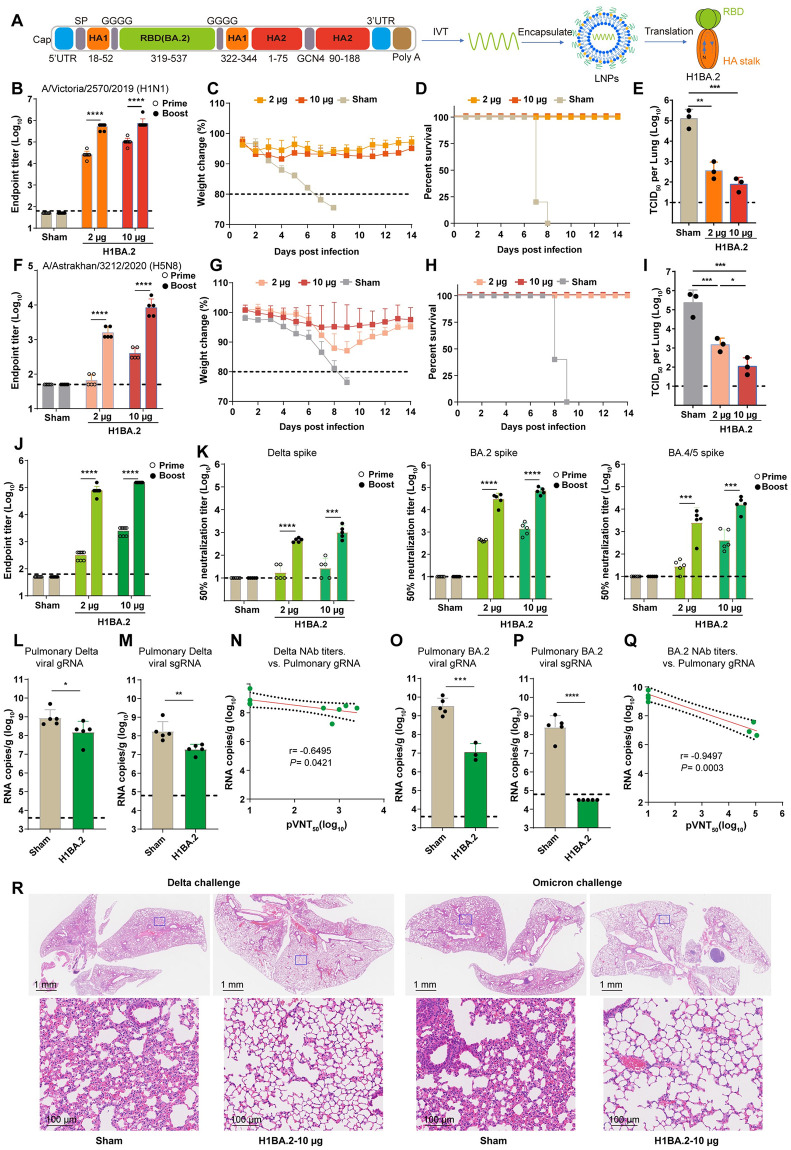
Development of H1BA.2 mRNA vaccine. (A) Schematic representation of the H1BA.2 vaccine design. SP, signal peptide. Groups of 6- to 8-week-old female BALB/c mice (n = 5) were vaccinated with two doses of H1BA.2 mRNA vaccine containing either 2 or 10 μg of immunogens at 3-week intervals. Serum samples were collected 19 and 35 days after the first immunization for ELISA and neutralization assays. (B) ELISA shows the H1 specific IgG titers. Mice were intranasally challenged with 20 × mLD_50_ of A/Brisbane/02/2018 (H1N1) virus at 63 days after the first immunization. Vaccine efficacy was assessed by measuring morbidity (weight loss) (C) and mortality (survival) (D) in five mice during the 14-day period. Lung tissues of the other three mice were harvested and split to test lung viral titers on 3 DPI (E). (F) ELISA shows the H5 specific IgG titers. Mice were intranasally challenged with 10 × mLD_50_ of reassortment A/Astrakhan/3212/2020(H5N8) virus at 78 days after the first immunization. Vaccine efficacy was assessed by measuring morbidity (weight loss) and (G) mortality (survival) (H) in five mice. Lung tissues of the other three mice were harvested and split to test lung viral titers on 3 DPI (I). (J) ELISA depicting the SARS-CoV-2 Delta RBD specific IgG titers. (K) Determination of 50% neutralization titer for pseudotyped virus (Delta, BA.2, and BA.4/5) in serum. Five mice were randomly selected from each group and challenged with 5 × 10^5^ TCID_50_ of Delta SARS-CoV-2 variant virus, and the other five were challenged with 5 × 10^5^ TCID_50_ of Omicron (BA.2) variant virus140 days after the initial immunization. Mice challenged with Delta variant had received Ad5-hACE2 intranasally 5 days before. All tests were done after 3 days post either Delta or Omicron variant infection. (L) Quantification of pulmonary Delta viral gRNA levels via qRT-PCR. (M) Quantification of pulmonary Delta viral sgRNA levels via qRT-PCR. (N) Plots show correlations and corresponding two-sided p values between pVNT_50_ of Delta variant and Delta viral gRNA. (O) Quantification of pulmonary Omicron viral gRNA levels via qRT-PCR. (P) Quantification of pulmonary Omicron viral sgRNA levels via qRT-PCR. (Q) Plots show correlations and corresponding two-sided p values between pVNT_50_ of Omicron variant and Omicron viral gRNA. (R) Histological pathology analyses of lung sections of mice challenged with Delta or Omicron. Shown are the representative lung sections by H&E staining. Scale bars are labelled.

In late 2021, the Omicron variant entered global circulation and gradually replaced its predecessor, Delta. In this context, we developed the H1BA.2 mRNA vaccine (Fig 6A). ELISA analysis showed that vaccine immunization effectively triggered the production of specific antibodies against influenza H1, H5 and SARS-CoV-2 RBD (Fig 6B, 6F and 6J). Pseudovirus neutralization experiments revealed that vaccination led to high titers of neutralizing antibodies against BA.2 and BA.4/5 and low titers against Delta pseudovirus (Fig 6K). Further influenza virus challenge experiments indicated complete protection against lethal challenges of influenza H1 and H5, as well as a significant reduction in viral load in the lungs of mice, thereby alleviating virus-induced lung damage (Fig 6C–6E and 6G–6I). For mice challenged with Delta variant, the averages of both gRNA (p = 0.04) and sgRNA (p = 0.006) were significantly reduced in immunized mice compared with the sham group (Fig 6L and 6M).

For mice challenged with Omicron variant, the sham group of mice developed high levels of pulmonary viral gRNA (average: 3.13 × 10^9^ copies/g) and sgRNA (average: 2.49 × 10^8^ copies/g) (Fig 6O and 6P). In contrast, mice vaccinated with the vaccine showed decreased pulmonary viral gRNA (average: 1.08 × 10^7^ copies/g) and undetectable sgRNA, suggesting the complete control of Omicron viral replication (Fig 6O and 6P). NAb titers inversely correlated with the reduction of pulmonary gRNA of both Delta variant (r = 0.6495, p < 0.0421) and Omicron variant (r = 0.9497, p = 0.0003) based on a linear model (Fig 6N and 6Q).

Histopathological analyses also indicated that sham mice had extensive lung damage with consolidated lesion and inflammatory cell infiltration across larger areas (Fig 6R). In contrast, the vaccine prevented tissue damage to a large degree, with only minor perivascular and alveolar infiltrates observed in very limited areas (Fig 6R).

## Discussion

Influenza viruses and SARS-CoV-2 pose significant threats to human health. The gradual adaptation and mutation of SARS-CoV-2 have led to increased transmissibility. Until now, SARS-CoV-2 was classified into 5 different serotypes based on RBD antigenicities [24–26], causing severe endemic epidemics worldwide. Meanwhile, influenza viruses have been seriously affecting human health for more than 100 years. Both viruses can cause respiratory disease, are highly mutable, and cases of co-infection between influenza and SARS-CoV-2 have been reported in recent years [27,28]. Therefore, annual vaccination may be required for future prevention and control. A two-in-one vaccine design for influenza virus and SARS-CoV-2 holds promising prospects, potentially simplifying the vaccination process and enhancing vaccination efficiency.

In previous studies, Deng *et al*. reported that an intranasal vaccine with live attenuated influenza virus (LAIV), which deleted the NS1 gene and encoded the SARS-CoV-2 S-RBD protein, induced high levels of neutralizing antibodies and T-cell responses in mice [29]. However, concerns arise due to the genetic reassortment nature of influenza virus, potentially leading to virulence enhancement. During the preparation of this manuscript, Wang *et al*. described a SARS-CoV-2 and influenza double hit vaccine based on RBD-conjugated inactivated IAV [30]. While commendable, this approach faces challenges linked to the intricacies of production and its limited spectrum of vaccine coverage. Moreover, there have been other designs using HA1 domain from IAV and RBD from SARS-CoV-2 [31], indicating the pursuit of a combination vaccine targeting both SARS-CoV-2 and IAV is a promising strategy to address the ongoing and potential future threats posed by these respiratory viruses. Both the live attenuated influenza virus-based approach and the RBD-conjugated inactivated influenza vaccine offer unique advantages and challenges. Additionally, there have been studies exploring the combined administration of developed mRNA or protein vaccines for influenza and COVID-19 [32,33]. This approach represents another strategy for multivalent vaccination. Therefore, it is crucial to continue exploring a broader-spectrum and highly effective influenza-COVID-19 combined vaccine.

Compared with other traditional vaccine platforms, mRNA vaccines have the advantages of safety, rapid, strong immunogenicity, and simple vaccine design and manufacturing [34], making them an attractive platform [35]. This is particularly valuable for emerging infectious diseases, such as a potential influenza pandemic or a variant of SARS-CoV-2. In this study, we developed a nucleic acid-modified mRNA vaccine (H1Delta vaccine) based on our previous design strategy of the chimeric protein vaccine for influenza and COVID-19 [13]. The mRNA vaccine was able to express the HA stalk and COVID-19 RBD chimeric antigen after entering cells. Animal experiment results showed that two doses of H1Delta vaccine immunized mice can induce strong humoral and cellular immunities against influenza virus and SARS-CoV-2. It is also able to provide complete protection against influenza viruses H1N1 and H5N8, as well as SARS-CoV-2 Delta and Omicron variants. Of particular note, the vaccine immunized mice developed high titer antibodies that lasted for at least 180 days and were fully protected against lethal doses of H1N1 challenge. With the continuous mutation of SARS-CoV-2, we then developed a vaccine (H1BA.2) targeting the Omicron variant, which can also provide protection against influenza virus and COVID-19 and has a better protective effect against the Omicron variant compared to the Delta variant.

Previous studies have shown that the antibody response induced by the HA stalk region is broadly protective and resistant to the lethal challenge of influenza viruses [36–38]. mRNA vaccines encoding the HA stalk region have also performed well [39–41]. Antibodies targeting the influenza stalk can play an antiviral role by blocking low-pH-induced HA conformational rearrangement, thereby preventing membrane fusion [42,43]. There is also growing evidence that the *in vivo* protection of antibodies against the influenza stalk region is largely mediated by antibody-dependent cellular cytotoxicity (ADCC) [40,44]. The mRNA vaccine immunization we constructed induced high titers of specific antibodies against influenza viruses H1N1 and H5N8, and the protective mechanisms may be related to these, which should be addressed in future studies. Recently, it has been reported that the titers of SARS-CoV-2 specific antibodies and neutralizing antibodies induced by RBD mRNA secondary immunization can reach 10^5^ and 10^4^, respectively [45]. Our results show that H1Delta vaccine immunologically induces higher SARS-CoV-2 specific antibody and neutralizing antibody titers, which may be due to the stronger immunogenicity of chimeric protein expressed by the vaccine compared to RBD monomer [46].

One of the advantages of mRNA vaccines is their ability to induce strong cellular immunity, which plays a crucial role in the clearance of multiple pathogens. In this study, vaccine immunization significantly increased the proportion of CD4^+^ and CD8^+^ spleen cells secreting IFN-γ and TNF-α, indicating a robust Th1-biased immune response. Meanwhile, we found that the level of cellular immunity against RBD was higher than that against the HA stalks. Consistent with previous studies [39,47,48], T cell immunity against influenza HA stalks is weaker, and this result raises the hypothesis that the protection against influenza virus challenge may be primarily mediated by antibodies. In contrast, T cell immunity plays a more important role in the protection against COVID-19 [49]. Several lines of evidence suggest that a robust cellular response can provide effective immune protection against COVID-19 [50–52]. CD4^+^ and CD8^+^ T cell responses can modulate disease severity in humans and inhibit viral replication in animal models [53–55]. Indeed, a study of mRNA-based T-cell-inducing antigens demonstrated that dual immunization with both these T-cell-inducing and RBD-based mRNA antigens was more effective in preventing SARS-CoV-2 infection in mice and non-human primates than vaccination with RBD alone [56].

Of particular note is the expansion of our vaccine design. In late 2021, the Omicron variant entered global circulation and gradually replaced its predecessor, Delta. We have developed a vaccine targeting the omicron variant strain, which can also provide protection against influenza virus and COVID-19. Notably, our findings show that the H1Delta vaccine produces antigens with higher immunogenicity, offering complete protection against the Delta variant challenge and fully inhibiting the replication of Omicron variant in the lungs. While the H1BA.2 vaccine provides good protection against the Omicron variant challenge, it does not offer fully protection against Delta variant challenge, consistent with the results observed in Qu *et al*.’s study [57].

There are several limitations to the study. Firstly, the protective efficacy of these two mRNA vaccines against SARS-CoV-2 Delta or Omicron variants was not evaluated in hamster or nonhuman primate models to obtain data more closely resembling human responses, due to limited availability of animals at the time of the study. Secondly, although the two-in-one mRNA vaccine strategy has shown cross-protective efficacy against different subtypes of IAVs or various SARS-CoV-2 variants, its breadth remains limited, unable to cover all viral strains. This necessitates further research, such as expanding design to encompass major clades of viruses, followed by combined administration or multivalent vaccination to enhance the vaccine’s broad-spectrum protection.

In conclusion, our results indicate that the excellent immunogenicity and scalability of H1Delta/H1BA.2 mRNA vaccine as a two-in-one vaccine for influenza and COVID-19 hold promising implications for the control of influenza epidemics and COVID-19 variants in the future.

## Materials and methods

### Ethics statement

This study was conducted in strict accordance with the recommendations of the Ethics Committee of the Institute of Microbiology, Chinese Academy of Sciences (IMCAS), in the Guide for the Care and Use of Laboratory Animals. All of the animal experiments were reviewed and approved by the Committee on the Ethics of Animal Experiments of IMCAS and the Ethics Committee of the National Institute for Viral Disease Control and Prevention, China CDC.

### Cells and viruses

Vero cells (ATCC), HEK293T cells (ATCC), Expi293F cells (ATCC) and Madin–Darby Canine Kidney (MDCK) cells (ATCC) were cultured at 37°C under 5% CO_2_ in Dulbecco’s modified Eagle’s medium (DMEM) supplemented with 100U/mL penicillin, 100 μg/mL streptomycin and 10% fetal bovine serum (FBS). All cell lines were tested negative for mycoplasma contamination.

A wildtype A/Brisbane/02/2018 (H1N1) virus (GISAID: EPI_ISL_522424), which exhibits a 99.7% sequence similarity with A/Victoria/2570/2019 in the HA stalk region, was used as the homologous H1 challenge virus. The heterosubtypic H5N8 challenge virus was a 6:2 re-assortment virus of A/Astrakhan/3212/2020(H5N8) (GISAID: EPI_ISL_1038924), in which HA and NA from A/Astrakhan/3212/2020(H5N8), in A/Puerto Rico/8/1934 (GISAID: EPI_ISL_22622) backbone. The two strains of influenza virus were propagated using 10-day-old SPF chicken embryos. Delta variant (NPRC 2.192100004) and Omicron variant (BA.2, NPRC 2.192100010) was propagated in Vero cells and titrated by TCID_50_ assay on Vero cells as previously described [46].

### Mice

Specific pathogen-free (SPF) female BALB/c mice were purchased from Beijing Vital River Laboratory Animal Technology Co., Ltd. (licensed by Charles River). They were housed under SPF conditions in the laboratory animal facilities at IMCAS and China CDC. Mice were housed with 5 companions per cage. The challenge studies with SARS-CoV-2 Delta and Omicron (BA.2) variants were conducted under animal biosafety level 3 (ABSL3) facility in China CDC. The age of mice at the time that experiments were performed were indicated in the corresponding Fig legends.

### Design and synthesis of nucleoside-modified mRNAs

mRNAs with modified nucleosides were constructed and synthesized as follows. Briefly, the RBD region of the SARS-CoV-2 Delta variant (GenBank: OK091006.1) replaced the head region of the A/Victoria/2570/2019 (H1N1) HA protein (GISAID: EPI1801581), while retaining the signal peptide (SP) of A/Victoria/2570/2019 (H1N1) HA. The two domains were joined by a linker (GSGS). The HA2 sequence at positions 76–89 was replaced with the GCN4 sequence (CMKQIEDKIEEIESK). Four mutations (K325C, V338K, I341K and R344Q) were introduced in HA1, and eight mutations (I10T, F63Y, V66I, K68C, F70Y, L73S, R76C and T93C) were introduced in HA2 to maintain the native conformation of the HA stalk [37]. Unlike the H1Delta protein vaccine, the mRNA vaccine was not designed with a six-histidine purification tag (His6-tag) [13]. The H1BA.2 design is similar to the H1Delta design, except that the SARS-CoV-2 Delta variant RBD is replaced by the BA.2 variant S protein RBD. The mRNAs contain a codon-optimized open reading frame flanked by a capped 5’UTR and 3’UTR and were transcribed *in vitro* using T7 high yield RNA transcription kit (Novoprotein, Shanghai, China) on linearized plasmids. mRNA is transcribed into a 120-nucleotide poly(A) tail and 1-methylpseudouridine -5′-triphosphate was used instead of UTP to generate modified nucleoside-containing mRNA during *in vitro* transcription to generate nucleotide-modified mRNA. mRNA was purified by overnight LiCl precipitation at -20°C, pelleted by centrifugation at 18,800 × g for 15 min at 4°C, washed with 70% ethanol, centrifuged at 18,800 × g for 5 min at 4°C, and resuspended in RNase-free water. Purified mRNA was analyzed by agarose gel electrophoresis and stored frozen at -80°C until use.

### mRNA vaccine production

mRNA was encapsulated in LNPs through a self-assembly process. Lipids were dissolved in ethanol at molar ratios of 50:10:38.5:1.5 (ionizable lipid: DSPC: cholesterol: PEG-lipid). The lipid mixture was combined with a 50 mM citrate buffer (pH 4.0) containing mRNA at a ratio of 3:1 (aqueous: ethanol) using a microfluidic mixer (Precision Nanosystems, Vancouver, BC) with a flow rate of 12 mL/min. Formulations were tested for particle size (ZetasizePro, Malvern Panalytical), RNA encapsulation (Quant-iT RiboGreen RNA Assay Kit, Invitrogen) [58] and polydispersity index (ZetasizePro, Malvern Panalytical). The mRNA-LNPs were stored at 4°C and the RNA concentration was about 1 mg/mL.

### mRNA transfection and Western blot

HEK293T cells were seeded in a 6-well plate and transfected with mRNA utilizing Lipofectamine MessengerMAX (Invitrogen) in accordance with the manufacturer’s instructions. After a period of 36–48 hours, the supernatant was harvested and combined with loading buffer containing dithiothreitol. Concurrently, the cells were lysed with Passive Lysis Buffer (Promega) and subsequently mixed with loading buffer containing dithiothreitol. The ensuing samples were subjected to SDS-PAGE for protein separation, followed by transfer onto a nitrocellulose membrane (Merck Millipore). The membrane underwent blocking using 5% non-fat milk diluted in TBS-T buffer, followed by incubation with anti-SARS-CoV-2 prototype RBD polyclonal antibodies for 1 hour. For detection, goat anti-rabbit-HRP IgG (Easybio) served as the secondary antibody. Post-incubation, the membrane was thoroughly washed and then developed utilizing Beyotime BeyoECL Plus (Beyotime Biotech) to visualize the protein bands. The mRNA encoding prototype RBD-dimer, previously reported by us, was employed as a positive control [52,59].

### Cryo-electron microscopy of LNPs

mRNA-LNPs sample was transferred onto a glow-discharged ultrathin carbon-coated copper grid (Zhongjingkeyi Company) followed by 60 s of waiting and then blotted for 2 s with filter paper before plugging into liquid ethane using the Vitrobot Mark IV. The frozen grids were transferred at liquid nitrogen temperature and loaded into a Talos 120c transmission electron microscope (Thermo Fisher Scientific) equipped with a field emission gun operated at 200 kV. The images were recorded on a direct electron detector (ED20) at a total electron dose of ~50e−/Å2.

### Immunization of mice with mRNA vaccine

Female BALB/c mice (six to eight weeks old) were immunized intramuscularly (i.m.) with 100 μL of mRNA-LNP or sham (control) on day 0 and day 21. Serum samples were collected at indicated times after vaccination, inactivated at 56°C for 30 min, and detected for antibody response and neutralizing antibodies as described below. Spleen tissues were collected at day 120 post initial immunization for evaluation of long-term cellular immune responses by ELISpot and flow cytometry as described below.

### Challenge of mice with H1N1 and H5N8

Two-to-four weeks after boost vaccination, mice were challenged intranasally (i.n.) with 50 μL of influenza A/Brisbane/02/2018 (H1N1) and A/Astrakhan/3212/2020 (H5N8) containing 20 × and 10 × the 50% mouse lethal dose (mLD_50_), respectively. Mice were monitored for 14 days to record body weight changes and survival rates following challenge. Weight loss of >20% was considered as the survival endpoint.

### Determination of lung influenza viral loads

The lung tissues collected at day 3 post-infection was homogenized, and influenza viral loads was determined by a TCID_50_ assay using MDCK cells. Briefly, MDCK cells were seeded in 96-wells flat-bottom plates and incubated overnight (37°C, 5% CO_2_). The cells were washed twice with PBS and then incubated 48h at 37°C with serial dilutions of the lung homogenates in quadruplicate in DMEM supplemented with 2 μg/mL of TPCK-treated trypsin. After 48 h, cells were fixed and permeabilized, followed by incubation with anti-NP antibody (Abcam, ab128193) and goat anti-mouse IgG(H+L) (HRP labeled) antibody. Viral titers were calculated as TCID_50_/mL according to the Reed-Muench method [60].

### Challenge of mice with Delta and Omicron SARS-CoV-2 variants

Mice experiments with Delta and Omicron variants challenge were conducted under ABSL3 facilities in CDC. The immunized mice were challenged intranasally with 5 × 10^5^ TCID_50_ of Delta variant or Omicron variant in a total volume of 50 μL under light anesthesia with inhaled isoflurane. BALB/c mice were anesthetized with isoflurane and intranasally transduced with 8 × 10^9^ vp of Ad5-hACE2 five days before Delta variant infection [23]. Mice were euthanized and necropsied at day 3, and lung tissues were collected for virus titration and pathological examination. SARS-CoV-2-specific qRT-PCR assays were performed using TaqMan Fast Virus 1-Step Master Mix kit (Thermo Fisher Scientific, USA) on a CFX384 Touch Real-Time PCR Detection System (Bio-Rad, USA) according to the manufacturer’s protocol. Two sets of primers and probes were used to detect a region of the N gene of viral genome (gRNA) [61] and a region of E gene of subgenomic RNA (sgRNA) of SARS-CoV-2 [62], respectively, with sequences as follows: gRNA-F, GACCCCAAAATCAGCGAAAT; gRNA-R, TCTGGTTACTGCCAGTTGAATCTG; gRNA-probe, ACCCCGCATTACGTTTGGTGGACC (Omicron variant gRNA-probe: ACTCCGCATTACGTTTGGTGGACC); sgRNA-F, CGATCTCTTGTAGATCTGT TCTC; sgRNA-R, ATATTGCAGCAGTACGCACACA; sgRNA-probe, ACACTAGCCATCCTTACTGCGCTTCG. Mice lung tissues were stained with H&E for pathological examination.

### Enzyme-linked immunosorbent assay

The HA or RBD recombinant proteins were expressed and purified *in vitro* using a mammalian expression system. The genes encoding the ectodomains of A/Victoria/2570/2019 (H1N1) and A/Astrakhan/3212/2020 (H5N8) were synthesized and cloned into pCAGGS vector in-frame with a His6-tag for purification. The genes encoding RBD of SARS-CoV-2 Delta variant and BA.2 variant were cloned into pCAGGS vector with a His6-tag at their C-terminal ends. Following transfection of Expi293F cells with polyethyleneimine (PEI), culture supernatants were collected after 5 days. Soluble proteins were recovered from cell supernatants by metal affinity chromatography using a HisTrap HP 5-mL column (GE Healthcare). The proteins were further purified by gel filtration chromatography using a Superdex 200 increase 10/300 GL column (GE Healthcare) with a running buffer of 20 mM Tris-HCl 150 mM NaCl (pH 8.0).

Influenza virus HA and SARS-CoV-2 RBD specific antibodies of different subtypes (IgG, IgG1, IgG2a) in serum were assessed by enzyme-linked immunosorbent assay (ELISA). Briefly, 96-well plates were coated overnight with 200 ng per well of HA or RBD recombinant protein in 0.05 M carbonate-bicarbonate buffer, pH 9.6. The following day, the coated plates were washed three times with PBS containing 0.1% Tween-20 (PBS-T). The plates were then blocked for 1 h at 37°C with 5% fat-free milk in PBS-T. Serum samples were twofold serially diluted and added to the first well at a final concentration of 1:100, and incubated on the plate for 1 h at 37°C. After washing three times with PBS-T, goat anti-mouse IgG-HRP (1:5,000), IgG1-HRP (1:5,000) or IgG2a-HRP (1:5,000) antibodies (Abcam) were added for 1 h at 37°C. Subsequently, the plates were washed three times with PBS-T and developed with 3,3′,5,5′-tetramethylbenzidine (TMB) substrate for 10 min. Reactions were stopped with 2 M H_2_S0_4_, and the absorbance was measured at 450 nm using a microplate reader (PerkinElmer, USA). Endpoint titers were defined as the highest reciprocal dilution of serum yielding an absorbance greater than 2.1 times the background values. Antibody titers below the limit of detection were assigned as one-third of the detection limit.

### Pseudotyped virus generation and neutralization assay

The VSV-ΔG-GFP based SARS-CoV-2 pseudotyped virus was constructed as previously described [63,64]. Briefly, the spike proteins with 18 amino acids deleted from the C-terminus of Delta, BA.2 and BA.4/5 SARS-CoV-2 variants were cloned into the pCAGGS vector, respectively. To optimize the plasmid for expression in mammalian cells, codon optimization was performed, and 30 μg of each plasmid was transfected into HEK-293T cells. 24 hours post-transfection, VSV-ΔG-GFP pseudotyped virus was added. The supernatant was then replaced with fresh complete DMEM medium containing anti-VSV-G antibodies (I1HybridomaATCC-CRL2700) after 2 hours. Following a 30-hour incubation at 37°C, the supernatants were collected, filtered through a 0.45 μm filter (Millipore), aliquoted, and stored at -80°C until further use.

Subsequently, the heat-inactivated (56°C for 30min) serum samples were serial diluted and incubated with SARS-CoV-2 pseudoviruses (1000 transducing units, TU) at a 1:1 ratio for 1h at 37°C. The mixture was transfer to pre-plated Vero cells in 96 well plates in 100 μL volume. The TU values were read on a CQ1 confocal image cytometer (Yokogawa) after a 15 h incubation. 50% neutralization titers were calculated with nonlinear regression (log[inhibitor] versus normalized response − variable slope) using GraphPad Prism 7.

### Enzyme-linked immunospot assay

To evaluate the cellular immune response stimulated by the vaccine, ELISpot assays were performed using IFN-γ or IL-4 precoated ELISpot kits (Dakewe Biotech), according to the manufacturer’s protocol. Briefly, splenocytes were added to the plates and stimulated with peptide pools (15 mers overlapping by 13 aa, 2 μg/mL each) covering A/Victoria/2570/2019 (H1N1) HA protein or SARS-CoV-2 RBD protein (Genscript) and cultured at 37°C with 5% CO_2_ for 20 hours. The cells were removed, and the plates were processed in turn with biotinylated detection antibody, streptavidin-ALP conjugate, and substrate. Equivalent 40% dimethyl sulfoxide (DMSO), the solution to dissolve the peptide pool, was used as the negative control. Phorbol 12-myristate 13-acetate (PMA)/ionomycin (Dakewe Biotech) was used as the positive control. The spots were counted and analyzed by using CTL-ImmunoSpot S5 (Cellular Technology Limited).

### Flow cytometric analysis of intracellular cytokines

Mouse splenocytes were added to the plate (1 × 10^6^ cells/well) and then stimulated with the peptide pool (2 μg /mL of individual peptide) for 4 h. 40% DMSO and PMA/ionomycin were used as the negative and positive control, respectively. The cells were incubated with Golgiplug (BD Biosciences) for an additional 12 h at 37°C. Cells were washed with FACS buffer and surface stains were performed with anti-mouse CD3/CD4/CD8 antibodies (BioLegend) for 30 min at RT. Cells were washed with FACS buffer, subsequently fixed and permeabilized in permeabilizing buffer (BD Biosciences). Intracellular stains were performed with fluorochrome-labeled anti-mouse IFN-γ/TNF-α/IL-2/IL-4/IL-5 antibodies (BioLegend) for 30 min at RT. LSRFortessa was used to acquire the flow cytometry data, which were then analyzed with FlowJo software.

### Histopathology assay

Lung tissues from mice were fixed in 4% neutral-buffered formalin, embedded in paraffin, and stained with hematoxylin and eosin (H&E). Images were captured using a LEICA Versa 200 and were processed using software K-Viewer1.5.5.8.

### Statistical analysis

All data plotted with error bars are shown as mean ± SEM except it is described differently. The P values were generated by analysis data with a two-tail unpaired t test using the Prism 7 program (GraphPad Software). For all figures, p values are represented by the following symbols: *p < 0.05, **p < 0.01, and ***p < 0.001.

## Supporting information

S1 Fig Characterization of mRNA-H1Delta vaccine. (A) *In vitro* expression of the mRNAs H1Delta and H1BA.2. Each mRNA was transfected into HEK293T cells. HEK293T cells were transfected with each mRNA, and the expression of antigens in the culture supernatants and cells was assessed using Western blot analysis with anti-SARS-CoV-2 prototype RBD polyclonal antibodies. The samples transfected with only Lipofectamine MessengerMAX was used as negative control (NC), and the samples transfected with mRNA encoding dimeric prototype RBD was used as positive control (PC) [52,59]. The Western blot confirmed the successful secretion of both H1Delta and H1BA.2 proteins with molecular weights of approximately 70 kDa (predicted molecular weights are around 53 kDa). (B) Measurement of particle size of mRNA-H1Delta LNPs by dynamic light scattering. (C) A representative cryo-electron microscopy image of mRNA-H1Delta LNPs solution following mRNA encapsulation. Scale bar, 100 nm.
(TIF)

S2 Fig A balanced Th1/Th2 immune response induced by H1Delta mRNA vaccine. Groups of 6- to 8-week-old female BALB/c mice (n = 8) were vaccinated with two doses of 2 or 10 μg H1Delta mRNA vaccine in 3-week intervals. Serum samples were collected 35 days after initial immunization. (A) ELISA shows the H1 specific IgG titers. (B) ELISA shows the H1 specific IgG1 titers. (C) ELISA shows the H1 specific IgG2a titers. (D) ELISA shows the SARS-CoV-2 Delta RBD specific IgG titers. (E) ELISA shows the SARS-CoV-2 Delta RBD specific IgG1 titers. (F) ELISA shows the SARS-CoV-2 Delta RBD specific IgG2a titers. LODs were indicated by dashed lines.
(TIF)

S3 Fig Robust cellular immune responses induced by H1Delta mRNA vaccine. (A) Representative images of ELISpot wells re-stimulation with H1 stalk peptide pools are shown. (B) Representative images of ELISpot wells re-stimulation with SARS-CoV-2 RBD peptide pool are shown. (C and D) Radar and stacked graphs showing the poly-functionality of the CD4^+^ and CD8^+^ T cell response specific to the H1 stalk, with geometric mean frequencies visualized. (E and F) Radar and stacked graphs showing the poly-functionality of the CD4^+^ and CD8^+^ T cell response specific to the SARS-CoV-2 RBD, with geometric mean frequencies displayed.
(TIF)

S4 Fig Pulmonary histopathology in mice vaccinated with H1BA.2 mRNA vaccine. (A) Shown are the representative lung sections from mice challenged with 20 × mLD_50_ of A/Brisbane/02/2018 (H1N1) virus at 3 DPI, stained with H&E. (B) Shown are the representative lung sections from mice challenged with 10 × mLD_50_ of reassortment A/Astrakhan/3212/2020(H5N8) virus at 3 DPI by H&E staining.
(TIF)

S1 Data Data that underlies this paper. (XLSX)
