# Supplementary figures and images for: A chimeric mRNA vaccine of S-RBD with HA conferring broad protection against influenza and COVID-19 variants
Source: PLoS Pathog. 2024 Sep 20;20(9):e1012508. doi: 10.1371/journal.ppat.1012508 (PMC11414905; doi:10.1371/journal.ppat.1012508)

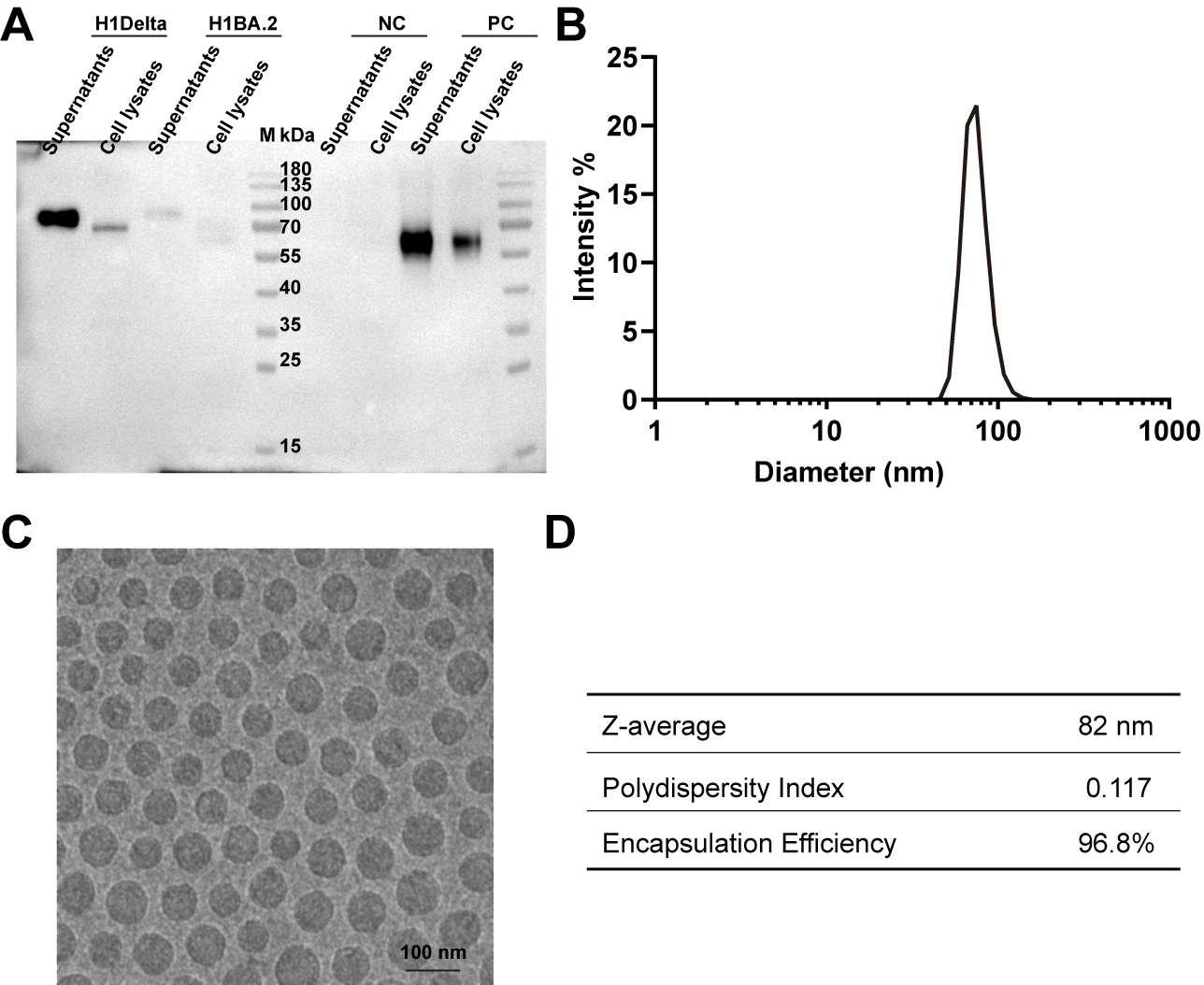

Supplement: S1 Fig — (A) In vitro expression of the mRNAs H1Delta and H1BA.2. Each mRNA was transfected into HEK293T cells. HEK293T cells were transfected with each mRNA, and the expression of antigens in the culture supernatants and cells was assessed using Western blot analysis with anti-SARS-CoV-2 prototype RBD polyclonal antibodies. The samples transfected with only Lipofectamine MessengerMAX was used as negative control (NC), and the samples transfected with mRNA encoding dimeric prototype RBD was used as positive control (PC) [52,59]. The Western blot confirmed the successful secretion of both H1Delta and H1BA.2 proteins with molecular weights of approximately 70 kDa (predicted molecular weights are around 53 kDa). (B) Measurement of particle size of mRNA-H1Delta LNPs by dynamic light scattering. (C) A representative cryo-electron microscopy image of mRNA-H1Delta LNPs solution following mRNA encapsulation. Scale bar, 100 nm. (TIF) [file ppat.1012508.s001.tif]

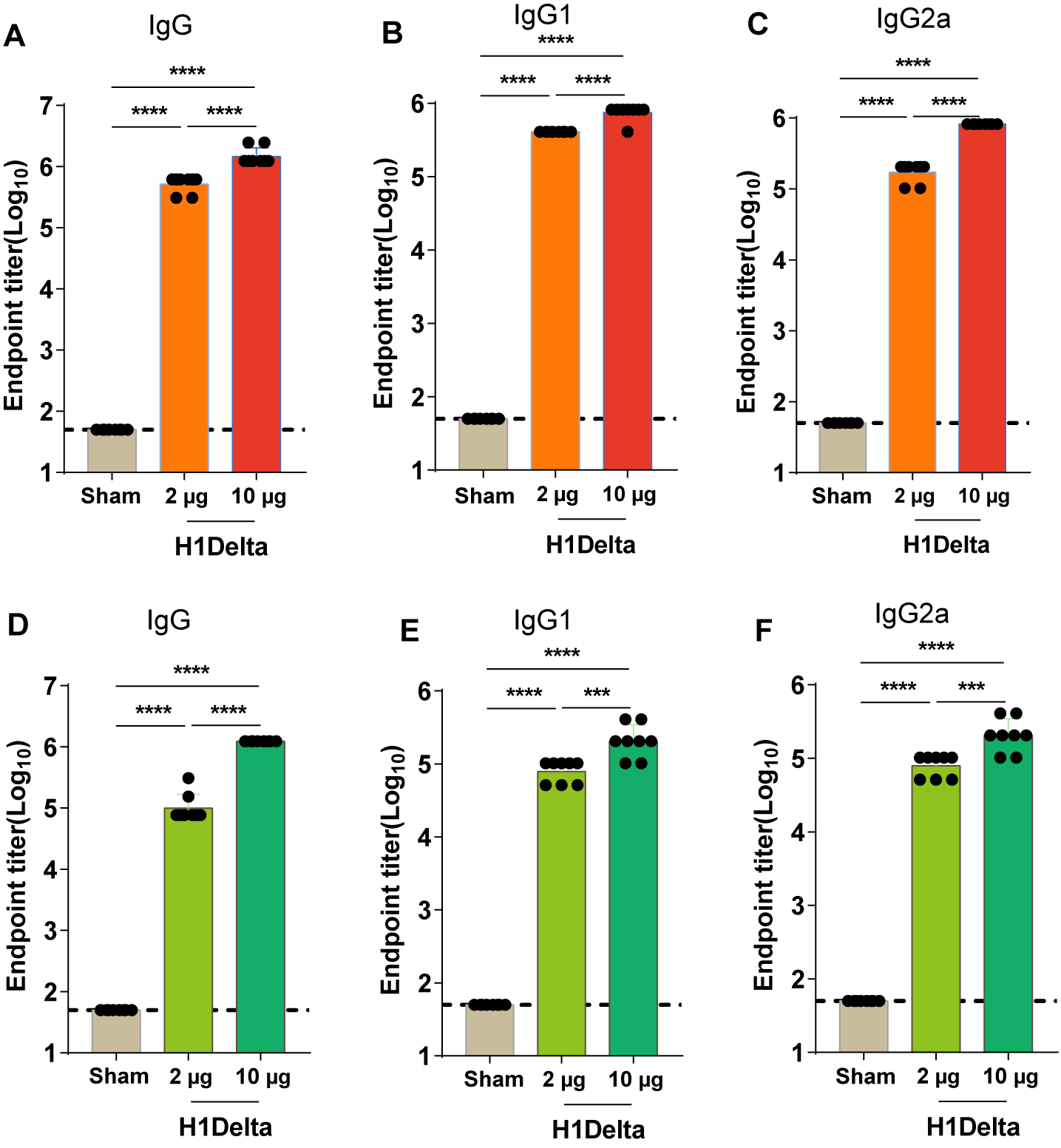

Supplement: S2 Fig — Groups of 6- to 8-week-old female BALB/c mice (n = 8) were vaccinated with two doses of 2 or 10 μg H1Delta mRNA vaccine in 3-week intervals. Serum samples were collected 35 days after initial immunization. (A) ELISA shows the H1 specific IgG titers. (B) ELISA shows the H1 specific IgG1 titers. (C) ELISA shows the H1 specific IgG2a titers. (D) ELISA shows the SARS-CoV-2 Delta RBD specific IgG titers. (E) ELISA shows the SARS-CoV-2 Delta RBD specific IgG1 titers. (F) ELISA shows the SARS-CoV-2 Delta RBD specific IgG2a titers. LODs were indicated by dashed lines. (TIF) [file ppat.1012508.s002.tif]

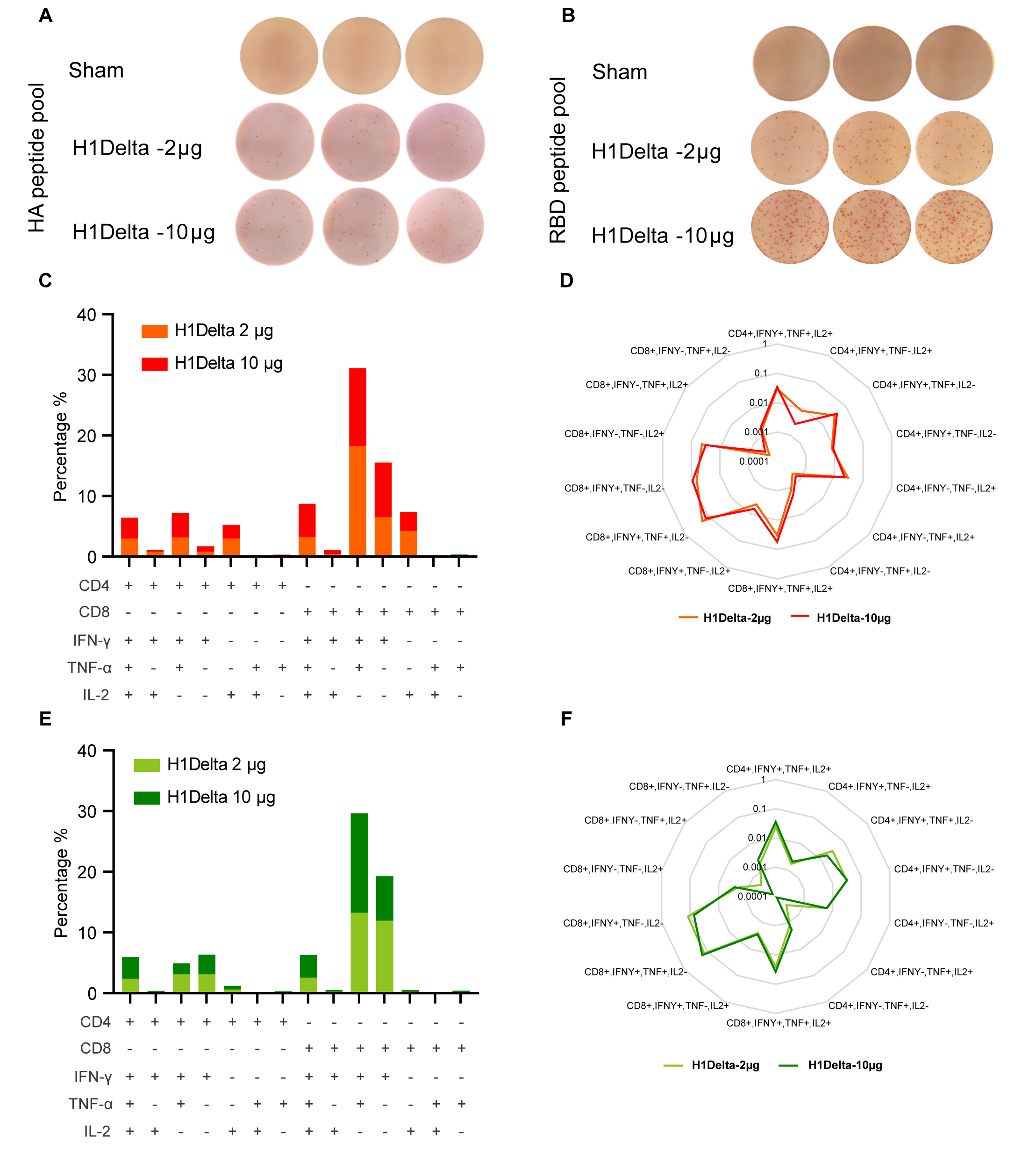

Supplement: S3 Fig — (A) Representative images of ELISpot wells re-stimulation with H1 stalk peptide pools are shown. (B) Representative images of ELISpot wells re-stimulation with SARS-CoV-2 RBD peptide pool are shown. (C and D) Radar and stacked graphs showing the poly-functionality of the CD4+ and CD8+ T cell response specific to the H1 stalk, with geometric mean frequencies visualized. (E and F) Radar and stacked graphs showing the poly-functionality of the CD4+ and CD8+ T cell response specific to the SARS-CoV-2 RBD, with geometric mean frequencies displayed. (TIF) [file ppat.1012508.s003.tif]

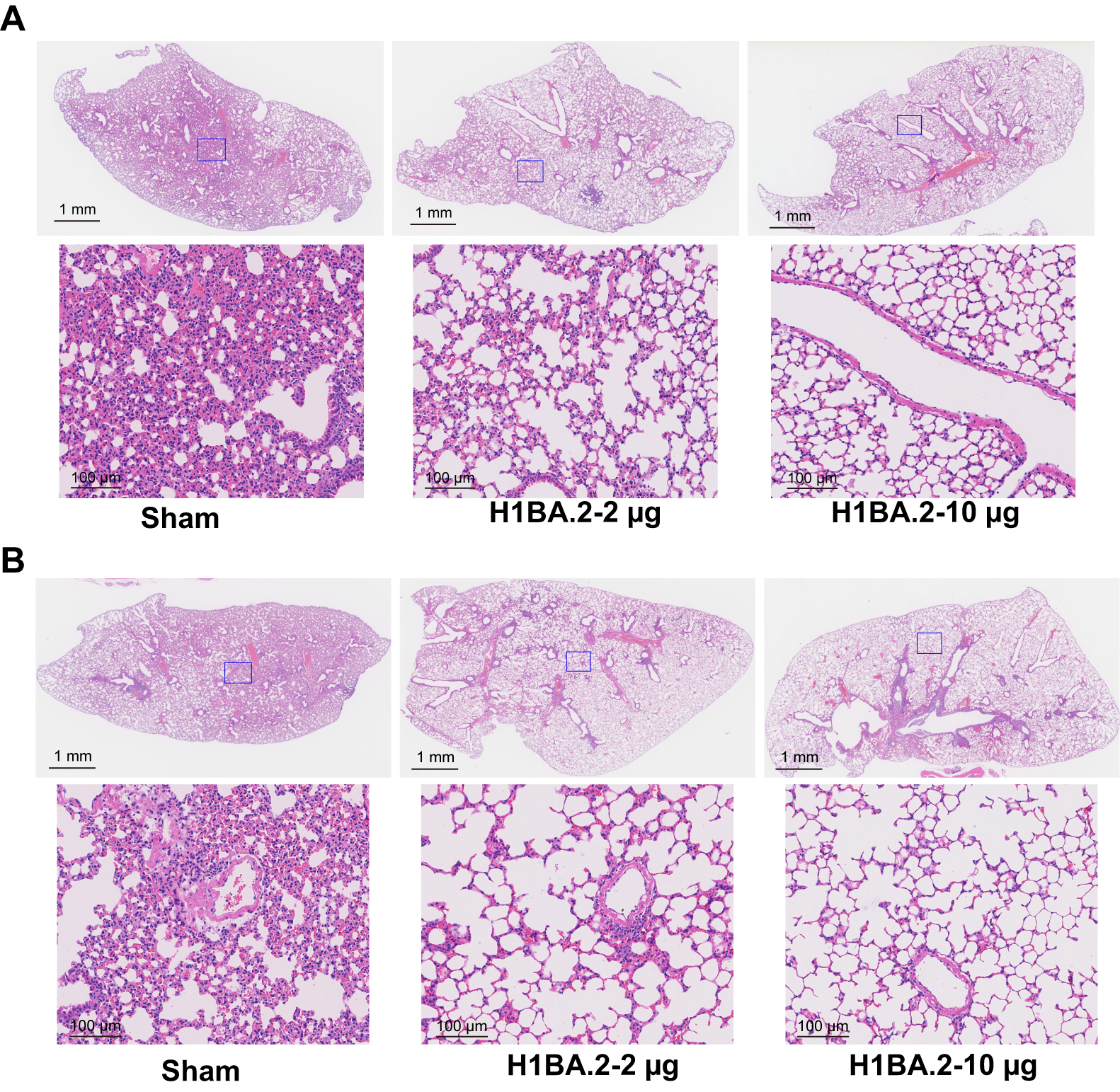

Supplement: S4 Fig — (A) Shown are the representative lung sections from mice challenged with 20 × mLD50 of A/Brisbane/02/2018 (H1N1) virus at 3 DPI, stained with H&E. (B) Shown are the representative lung sections from mice challenged with 10 × mLD50 of reassortment A/Astrakhan/3212/2020(H5N8) virus at 3 DPI by H&E staining. (TIF) [file ppat.1012508.s004.tif]
